# Development of a thermal model for irreversible electroporation: an approach to estimate and optimize the IRE protocols

**DOI:** 10.1007/s11548-021-02403-3

**Published:** 2021-05-25

**Authors:** Girindra Wardhana, João Pedro Almeida, Momen Abayazid, Jurgen J. Fütterer

**Affiliations:** 1 Department of Robotics and Mechatronics, The Faculty of Electrical Engineering, Mathematics and Computer Science, Technical Medical Centre, University of Twente, 7522 NB Enschede, The Netherlands; 2 Department of Medical Imaging, Radboud University Medical Center Nijmegen, Geert Grooteplein Zuid 10, 6525 GA Nijmegen, The Netherlands

**Keywords:** Irreversible electroporation, Finite element method, Interventional procedures, Modeling

## Abstract

**Purpose:**

Irreversible electroporation (IRE) is an emerging technique that has drawn attention in the field of cancer treatment. IRE uses non-thermal electric pulses to induce death of cancerous cells. However, recent studies have shown that the application of this technique may result in heating of the tissue. There is still room for improving its efficiency and defining better treatment protocols. This study investigates the optimal IRE protocols that avoiding the thermal damage during the IRE treatment.

**Methods:**

Electrode and pulse parameter are investigated. Finite element models are created to evaluate the ablation area and the temperature changes in the tissue. The model is validated experimentally in bovine liver tissue, while the parameters were optimized using response surface method (RSM).

**Results:**

From analysis of variance, the parameter of electrode distance and input voltage has significant effect to the temperature rise in the IRE treatment of bovine liver (*P* = 0.020 and *P* = 0.003 respectively). Meanwhile, only the input voltage significantly affects the ablation area (*P* < 0.001). The optimal result from RSM showed that for maximum ablation area 250.82mm^2^ with no thermal damage, the IRE protocol consisted of an active electrode length of 10 mm, a distance between electrodes of 10 mm, and the delivery of 50 pulses of 41.21 µs and 3000 V.

**Conclusions:**

The approach presented in this study allows the optimization of the IRE protocols. An optimal IRE protocol that maximizes the ablation area was successfully calculated which can be applied with no risk of thermal damage to the tissue.

## Introduction

Pancreatic adenocarcinoma is the most common malignancy of the pancreas and it is the fourth leading cause of cancer death in men and women [1, 2]. This cancer commonly metastasizes to other organs, especially to the liver [3]. During diagnosis, liver metastases are detected within more than 50% of the patients with pancreatic cancer [4]. The available options for the treatment of liver metastases are scarce, with surgical resection remaining as the common curative method [5]. However, not all the patients are appropriate for this type of treatment [6]. In addition, due to the late diagnosis, most patients present tumors in an advanced stage and resection is no longer possible [5].

Irreversible Electroporation (IRE) is a minimally invasive surgical procedure that has drawn interest in the field of cancer ablation over the last decade. The electroporation technique consists in the exposure of cells to strong electric fields delivered by electrodes inserted in the soft tissue. If the applied electric field is strong enough, electroporation can be irreversible, which is characterized by the irreversible generation of nanopores in the plasma membrane leading to eventual cell death [5, 7]. Theoretically, the process of tissue ablation generated by IRE can be assumed as non-thermal since it relies on electrical energy to disrupt the cell membrane [5]. However, if the parallelism of the needles is not maintained during the treatment, the temperature may increase as describe by Van Den Bos et al. [8]. In addition, recent studies also shown that the application of this technique may result in heating of the tissue [9, 10]. During IRE, some of the electrical energy that is delivered to the cells is usually converted into thermal energy, increasing the temperature. If it exceeds a certain threshold, undesired thermal damage of the healthy tissue may occur instantaneously [11]. The rate of damage drastically increases around 50–60 °C [12], where Arrhenius equation was normally used to measure the amount of the thermal damage [13].

Several parameters can have an influence on the outcomes of an IRE treatment. These parameters establish the IRE protocols of the treatment, and they are mostly related to the pulses that are delivered to the cells (pulse parameters) and to the configuration of the inserted electrodes (electrode parameters). The electric field generated in the tissue and the consequent temperature results change depending on the applied configuration. As a result, by adjusting some of these parameters, the thermal effect caused by heating can be reduced without compromising the ablation process.

Optimization of the IRE protocols is therefore essential to avoid the thermal damage that may occur on vital structures near to the liver, such as vasculature, gall bladder, or bile duct [14]. Nevertheless, the optimal combination of parameters is still unknown, whereby there is still room for improving the efficiency of the IRE method. Thus, one question stands out: *what can we do to optimize the outcomes of an IRE procedure?*

To the best of our knowledge, there are still undefined guidelines and uncertainty about the ideal IRE protocols that should be applied to a specific treatment, particularly related to combination of pulse and electrode parameters configuration. As a result, this paper presents an approach that allows the calculation of optimal IRE protocols by developing an IRE model with experimental validation. The final goal is to present the optimal IRE protocols that maximizing the ablation area and avoiding the thermal damage during the IRE treatment.

## Related work

This section presents published studies related to IRE and the corresponding IRE parameters in relation to the electric distribution and the thermal development during the IRE treatment. At the end of this section, the approach proposed in this study is described.

During IRE treatment planning, multiple parameters are considered to determine the ablation zone, including the electrode and pulse parameter. In terms of the electrode parameters, several studies have been done to investigate their effect on the IRE treatment. Davalos et al. [15] demonstrated that the electrode diameter had an effect on the maximum voltage that can be applied to the tissue before the temperature reaches 50 °C. They also showed that the electrode shape affects the electric field distribution and revealed that spherical electrodes showed less time to reach 50 °C than cylindrical electrodes [16]. Yang et al. studied the influence of four electrode properties (diameter, length, distance between electrode, and electrode number) on the volume of ablated tissue and maximum temperature generated in a liver tissue [17]. The result showed that only the distance between the electrode and the electrode number has significant effect to the maximum temperature, while only the electrode length significantly affects the ablation volume.

Regarding the pulse parameter, Garcia et al. [11] investigated the effect of pulse number to the probability of cell death due to IRE and thermal damage in a 2D liver model using bipolar electrode. Results from the simulations showed that at 30 pulses thermal damage starts occurring around the electrodes and that at 90 pulses, there is already significant damage in the tissue. Wandel et al. [18] characterized the effect of pulse number and pulse width in a porcine model. It is found that the higher pulse number and greater pulse width can increase the ablation zones.

Although several investigations have been done related to the electrode and pulse parameter in IRE, much of the research only considered them separately, despite the fact that both parameters need to be planned together. It is still not known the optimal configuration for both electrode parameter and pulse parameter in order to obtain the maximum ablation area with no thermal damage.

The present research contributes to the development of an experimentally validated model to adjust the IRE protocols that related to electrode and pulse parameter, and thus give guidance to the operator regarding the optimal configuration needed. An experimental apparatus is developed to validate finite element models for IRE simulation. A set of experiments was designed and performed in ex vivo bovine liver tissue to validate the models. In these experiments, temperature measurements were taken and then compared to the results obtained from simulations. If the temperature values measured experimentally are similar to the values calculated from the models, then they can be assumed as a reliable representation of the real phenomena. After validation, the models will be used to find the optimal IRE parameter configuration using response surface methodology (RSM).

## Methods

This section presents the developed models to simulate IRE and the experiment setup and procedure that were performed to validate the models.

### Experimental setup

**Fig. 1:**
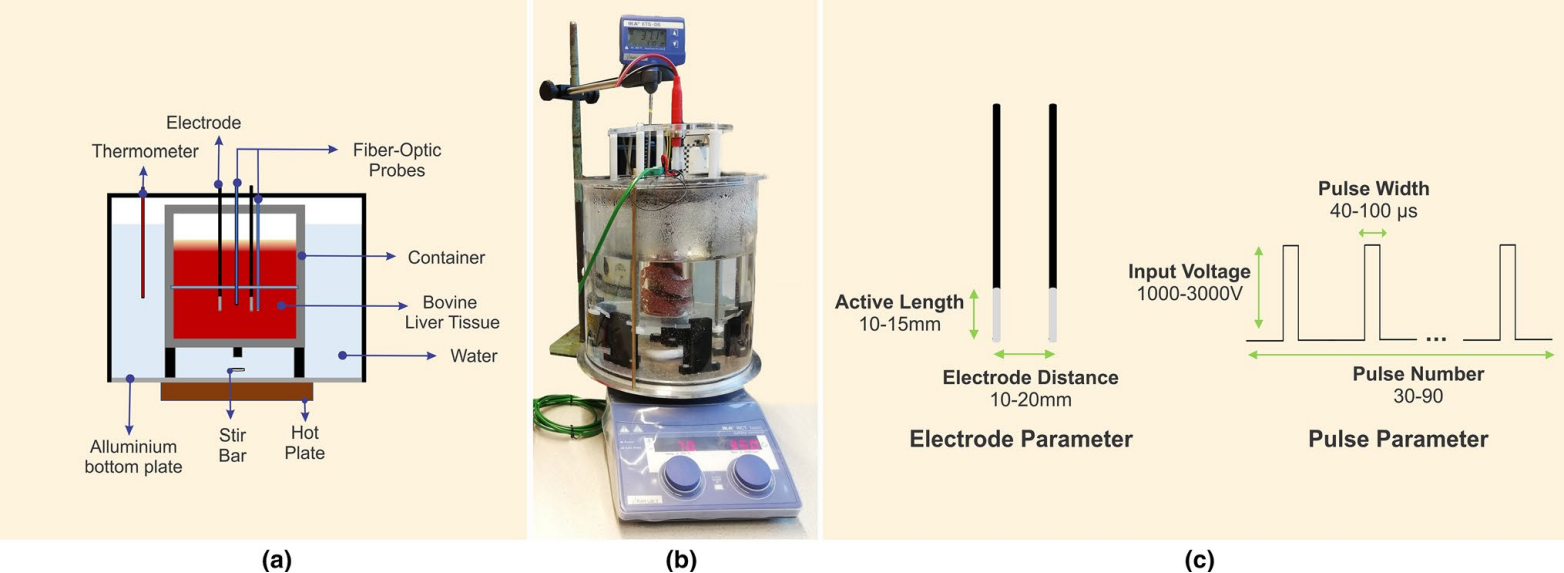
**a** Schematic representation of the experiment setup for model validation. Temperatures from three different location were measured, where location between electrodes and outside the electrode using fiber-optic probes, and water temperature using thermometer. **b** Actual experiment setup. **c** Five IRE parameters that were tested in the experiment

A schematic representation of the experimental setup is presented in Fig. 1a. The experimental setup consists of a transparent cylindrical container made of polymethyl methacrylate (PMMA) (40 × 150 mm). Bovine liver was purchased from a local butcher store in a fresh condition within 24–48 h after the animal was slaughtered. The bovine liver tissue did not go through freezing, and thus we expect that the conductivity will not be significantly affected. It was used directly for the experiment after we cut it into smaller tissue samples. They were put inside the container that was placed in a thermostatic bath at a controlled temperature of 37 °C in order to mimic the body temperature. A pulse generator system Gemini X2 (BTX, Holliston, MA) was connected to two stainless-steel cylindrical electrodes inserted in parallel into the bovine liver tissue sample and delivered a train of square pulses. A high-voltage probe (BTX Enhancer 3000) measured the amplitude of the delivered pulses and displayed the output in a digital oscilloscope RTB2004 (Rohde & Schwarz, Munich, Germany).

Fiber-optic temperature measurement probes “T1 Fiber Optic Temperature Probe” (Neoptix, Québec, Canada) were inserted in the liver tissue to measure the temperature between the two electrodes. Another fiber-optic probe was inserted in the bovine liver tissue, 3 mm away from the right electrode and at the same depth of the tip of the electrodes, in order to monitor the temperature outside the electrodes. Fiber-optic probe was chosen to measure the temperature due to its invulnerability to the electromagnetic interference that may occur around the electrode. The actual experiment setup is shown in Fig. 1b.

### Design of the experiment

Five IRE parameters were tested in different levels. Two cylindrical electrodes were inserted into biological tissue. The distance between electrodes, the applied voltage, the number of pulse repetitions and the pulse width were evaluated in three levels (low, medium, and high). Meanwhile, the active length of the electrodes was tested in two different levels (low and high). The different levels of the parameters in this study present in Table 1 and Fig. 1c. The frequency of the pulse delivery was defined as 1 Hz.

**Table 1:** Various IRE parameters with different configuration level

Level	Active length (mm)	Distance between electrodes (mm)	Input voltage (V)	Pulse repetitions	Pulse width (µs)
1	10	10	1000	30	40
2	15	15	2000	60	70
3	–	20	3000	90	100

### Finite element models

Finite element models were created in order to analyze the effect of the IRE parameters in the electric field and temperature responses. The computation of the finite element models was performed using COMSOL Multiphysics v.5.4. The geometry consists of a 2D longitudinal cut perpendicular to the two electrodes inserted into bovine liver tissue. Finite element method (FEM) was used to solve the mathematical models. The mesh was built automatically by COMSOL with a custom size for each domain. For liver and electrodes domain, maximum element size was set to 3.3 mm and 1 mm, while minimum element size was 3.1 mm and 0.8 mm, respectively. The elements had a triangular shape, where the number of elements for each configuration of electrodes is presented in Table 2.

**Table 2:** The number of elements for each electrode configuration

Distance between electrodes (mm)	Active length (mm)	Number of elements
10	10	16,974
10	15	17,160
15	10	18,481
15	15	18,579
20	10	19,440
20	15	19,600

The electric field distribution in the liver was determined by the Laplace equation:

1 $$ \nabla^{2} \cdot V = 0. $$

To model heat transfer within the biological tissue, the Bioheat Transfer Equation was considered:

2 $$ \rho c_{p} \frac{\partial T}{{\partial t}} + \rho c_{p} u \cdot \nabla T + \nabla \cdot q = Q_{s} + Q_{{{\text{bio}}}} $$

where $\rho$, $c_{p}$ and $T$ are the density, heat capacity and temperature of the tissue, respectively, and $q$ the heat flux by conduction in the tissue. $Q_{{\text{s}}}$ is the energy source term, sometimes mentioned as specific absorption rate (SAR) in the literature [19]. $Q_{{{\text{bio}}}}$ is the bioheat term that contains the perfusion source term $Q_{{{\text{bl}}}}$ and the metabolic heat generation term $Q_{{{\text{met}}}}$:

3 $$ Q_{{{\text{bio}}}} = Q_{{{\text{bl}}}} + Q_{{{\text{met}}}} $$

4 $$ Q_{{{\text{bl}}}} = \rho_{{\text{b}}} c_{{p,{\text{b}} }} \omega_{{{\text{b}} }} \left( {T_{{\text{b}}} - T} \right) $$

where $\rho_{{\text{b}}}$ is the blood density, $c_{{p,{\text{b}}}}$ the specific heat of blood, $\omega_{{\text{b}}}$ the blood perfusion rate, $T_{{\text{b}}}$ the arterial blood temperature and $T$ the temperature in the tissue.

To fully define the model, boundary conditions such as electrical insulation and thermal insulation of the electrodes were established. The liver tissue was also electrically insulated from the external environment. The initial temperature of the tissue was set at 37 °C. Table 3 presents the terms used to represent blood perfusion and metabolism to model heat transfer in biological tissue.

**Table 3:** Bioheat properties from blood and metabolism for heat transfer model in biological tissue

Parameter	Symbol	Unit	Value	References
Blood density	$\rho_{{\text{b}}}$	Kg/m^3^	1000	[19]
Blood temperature	$T_{{\text{b}}}$	°C	37	–
Blood specific heat capacity	$c_{{p,{\text{b}}}}$	J/(Kg∙ °C)	3640	[19]
Blood perfusion rate	$\omega_{{\text{b}}}$	1/s	5e^−4^	[19]
Metabolic heat source	$Q_{{{\text{met}}}}$	W/m^3^	0	[20]

The electrical and thermophysical properties of the bovine liver tissue were included into the model and they are summarized in Table 4. The relative permittivity $\varepsilon_{{{\text{r}},{\text{bov}}}}$ and thermal conductivity $k_{{{\text{bov}}}}$ were set according to their changes with temperature, and electrical conductivity $\sigma_{{{\text{bov}}}}$ in the function of electric field.

**Table 4:** Electrical and thermophysical properties of bovine liver tissue

Parameter	Symbol	Unit	Value	References
Density	$\rho_{{{\text{bov}}}}$	Kg/m^3^	1050	[21]
Heat capacity	$c_{{p,{\text{bov}}}}$	J/(Kg °C)	3400	[22]
Relative permittivity	$\varepsilon_{{{\text{r}},{\text{bov}}}}$	–	(See Fig. 2a)	[21]
Thermal conductivity	$k_{{{\text{bov}}}}$	W/(m °C)	(See Fig. 2b)	[23]
Electrical conductivity	$$\sigma_{{{\text{bov}}}}$$	S/m	$$\sigma_{{{\text{MIN}}}} :0.0650$$$$ \sigma_{{{\text{MAX}}}} :0.1483$$ (See Fig. 2c)	[24]

**Fig. 2:**
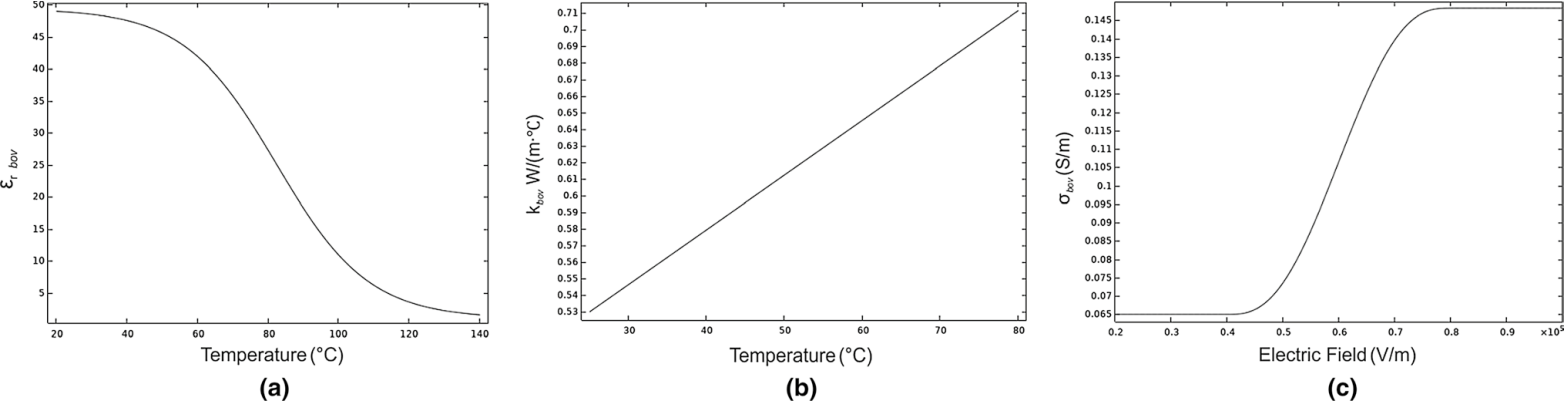
**a** Relative permittivity and **b** thermal conductivity in the function of temperature, and **c** electrical conductivity in the function of electric field for bovine liver tissue

## Results

In this section, the results of the experiments performed to validate the models are presented. After model validation, the optimization process to obtain the optimal IRE parameter configuration for bovine liver is also described.

### Validation of the models

**Fig. 3:**
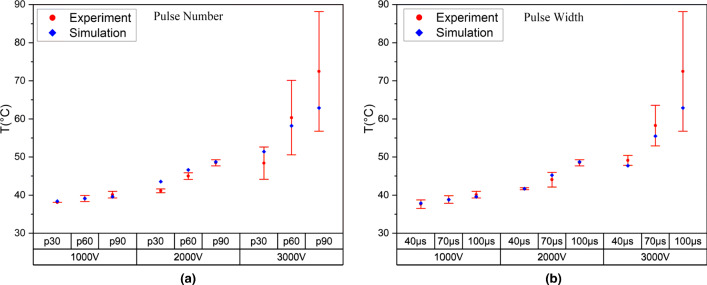
Maximum temperature in the IRE experiment and models simulation in bovine liver tissue with varying **a** pulse number and **b** pulse width

Finite element model was empirically validated by comparing the model result with the temperature measured in the bovine liver tissue during the IRE experiments. The experimental results are presented in Fig. 3. Three trials of experiments were performed for each combination, where active length and distance between electrode were set to constant at 10 mm.

Figure 3a presents the temperature achieved by varying the pulse number and the voltage for each experiment. Pulse width was set to constant of 100 μs while combination of pulse number 30, 60, and 90, and input voltage 1000 V, 2000 V, and 3000 V were used. The errors of the temperature measured in the center of the electrode were relatively low, around 3.9 ± 4.2%, except for the last experiment with 3000 V and 90 pulses that achieved error up to 13.2%. Regarding the thermal damage, bovine liver tissue reached maximum temperature more than 50 °C, when the voltage was set to 3000 V and pulse number 60 and 90. Surprisingly, the figure also revealed that with an increase in pulse number and voltage, the maximum temperature in bovine liver was also increased.

The result from the experiment with the pulse width is shown in Fig. 3b. In this experiment, pulse number was set to 90 pulses, while the pulse width and voltage were set to be varied. The error percentage for all the experiment was around 2.9 ± 4.2% for the temperature measurement except for the last experiment with 3000 V and 100 μs combination. Overall, the temperature between the FEM simulation and the ex vivo experiments was quite similar, and it is possible to affirm that the models were validated and can be used for calculating the response of the IRE parameters in the optimization study.

### IRE optimization for the treatment on liver tissue

According to Table 1, the experiments will have 162 different possible combinations of parameters. To reduce the number of experiments to be performed without losing significant information, the Taguchi method was used [25]. An L18(2^1^ 3^4^) Taguchi design was applied using Minitab 18, resulting 18 combinations of parameters.

After validating the models, the IRE experiment using 18 combination of five IRE parameters was performed by using the FEM analysis. The response of these IRE parameters to the maximum temperature and ablation coverage area (EF > 800 V/m) are presented in Table 5.

**Table 5:** Ablation area and maximum temperature response on various IRE parameter configuration

Experiment number	Active length (mm)	Distance between electrodes (mm)	Input voltage (V)	Pulse number	Pulse width (µs)	Ablation area (mm^2^)	Maximum temperature (°C)
1	10	10	1000	30	40	68.79	37.58
2	10	10	2000	60	70	135.56	43.67
3	10	10	3000	90	100	216.57	62.90
4	10	15	1000	30	70	9.46	37.00
5	10	15	2000	60	100	192.34	42.29
6	10	15	3000	90	40	297.33	43.34
7	10	20	1000	60	40	1.35	37.00
8	10	20	2000	90	70	208.79	39.82
9	10	20	3000	30	100	330.24	42.21
10	15	10	1000	90	100	95.03	39.66
11	15	10	2000	30	40	157.81	39.53
12	15	10	3000	60	70	237.69	52.61
13	15	15	1000	60	100	10.50	37.30
14	15	15	2000	90	40	216.62	39.88
15	15	15	3000	30	70	313.66	43.27
16	15	20	1000	90	70	4.74	37.35
17	15	20	2000	30	100	215.80	39.34
18	15	20	3000	60	40	375.46	40.61

**Fig. 4:**
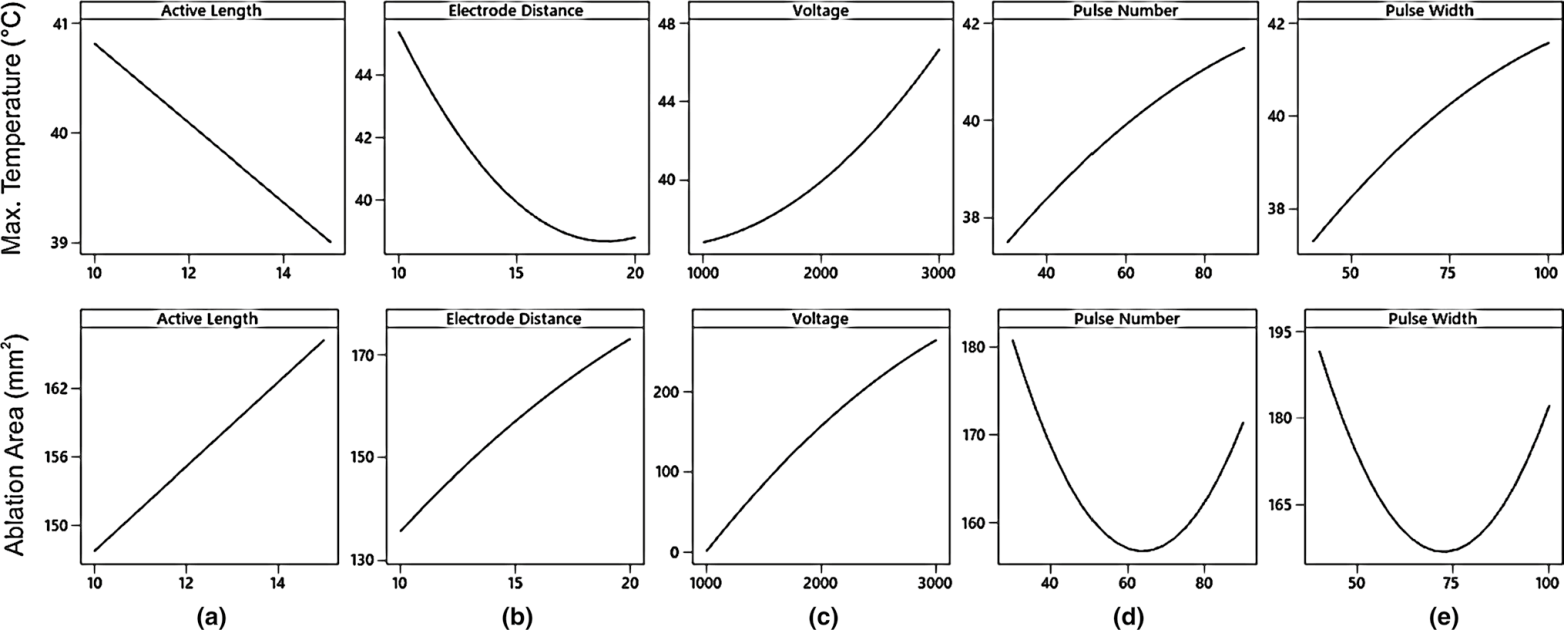
Maximum temperature and ablation coverage area (EF > 800 V/s) response to various IRE parameters configuration, including: **a** active length, **b** electrode distance, **c** voltage, **d** pulse number, and **e** pulse width

**Fig. 5:**
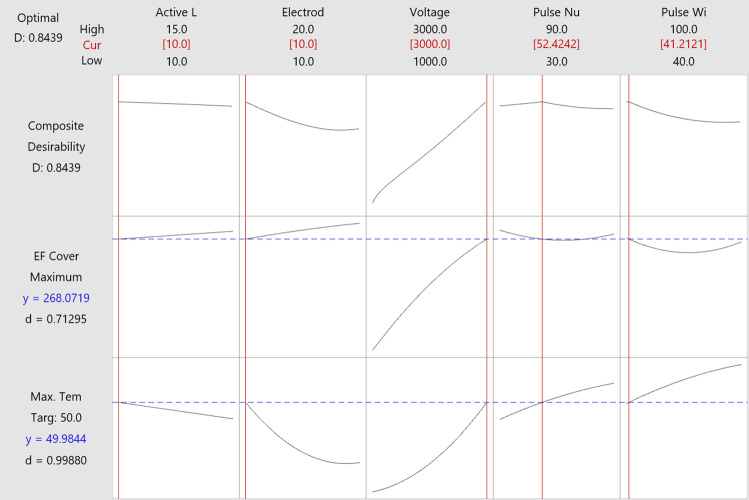
Optimization results obtained from RSM. The optimal parameters are presented on the top row, between brackets and in red. The red lines in the graph represent the optimal solutions. The blue dashed lines represent the target values of the objective functions, in this case, the maximum electric field and a temperature of 50 °C

RSM was used in Minitab to analyze the result in Table 5. From this method, the relationship between the responses and the variables can be obtained in the form of factorial plots as seen in Fig. 4. Also, the optimal solution of the IRE parameter configuration can be calculated as seen in Fig. 5, to obtain the maximum ablation area between the electrodes without achieving thermal damage in the tissue (temperature lower than 50 °C).

Based on the result in Fig. 4a, b, increasing the active length and the distance of the electrodes is the best option to reduce the maximum temperature while enlarging the ablation area during the treatment. More significant ablation area can be achieved by adjusting the voltage, but it has to be chosen carefully since it also increases the temperature considerably according to Fig. 4c. In term of pulse number (Fig. 4d) and pulse width (Fig. 4e), it is better to use a smaller value to keep the temperature low. Moreover, no significant result can be seen from increasing pulse number and pulse width to the effect of ablation area.

From analysis of variance, the parameter of electrode distance and input voltage has significant effect to the temperature rise in the IRE treatment of bovine liver (*P* = 0.020 and *P* = 0.003 respectively). Meanwhile, only the parameter of input voltage significantly affects the ablation area (*P* < 0.001)*.*

From Fig. 5, the optimal IRE parameter configuration calculated by RSM consists of the insertion of two needle electrodes with an active length of 10 mm and separated by 10 mm, and the delivery of 52.42 pulses with a width of 41.21 µs and amplitude of 3000 V. The number of pulse repetitions was defined as 50 instead of the result of 52.42 calculated by RSM.

The optimal parameters obtained from RSM were inserted in the models to assess the reliability of the method. The ablation area and the maximum temperature achieved at the center point between the electrodes were measured. The outcomes of each response were compared with the ones obtained with RSM by calculating the relative error. The results are shown in Table 6.

**Table 6:** Comparison of the optimization and simulation results with the optimal IRE parameter configuration for liver tissue

Response	RSM	Models	Error (%)
Area (mm^2^)	268.07	250.82	6.43
Maximum Temperature ( °C)	49.98	45.36	9.24

There was a good agreement between the electric field calculated by RSM and by the models. Regarding the temperature, RSM produced an overestimated value. The maximum temperature obtained from the simulation was 45.46 °C, which is less than the threshold for thermal damage (50 °C). Therefore, the error is not significant, since it is less likely that thermal damage occurs in the tissue.

### Electric field and temperature distributions for the optimal IRE parameter configuration

**Fig. 6:**
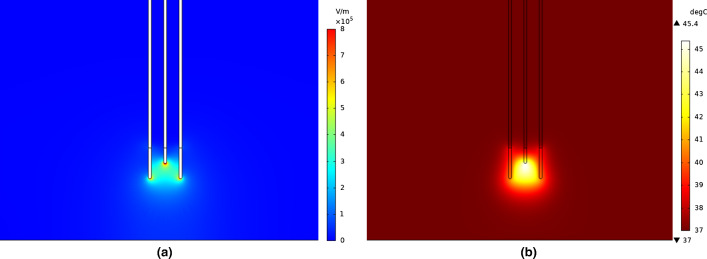
**a** Electric field distribution and **b** temperature distribution in liver tissue when applying the optimal IRE configuration (best view in color)

Once verified the reliability of the optimization process, the electric field and temperature distributions on liver tissue were calculated using simulations. The graphical representation of the simulation result for the electric field and temperature distribution is displayed in Fig. 6.

The electric field distribution between the electrodes seems to be quite homogeneous. However, in Fig. 6a, one can notice that the intensity of the generated electric field is higher near the corners of the electrodes. Electric charges tend to spread as much as possible on the surface of a conductive material, and, therefore, there is a higher concentration of charges in the tips of the electrodes.

The temperature increase is more substantial in the area between the electrodes, and it follows a pattern similar to the electric field distribution as shown in Fig. 6b. Here, the temperature seems to achieve its highest values near the vicinity of the electrodes and then eventually decreases with distance. The increase in temperature outside the area between the electrodes is not significantly high as in between them. Overall, no thermal damage is expected to occur in the tissue when applying the calculated optimal IRE parameter configuration.

## Discussion

This study presented a statistically based (Taguchi, RSM) approach to calculate optimal IRE parameter configuration taking into account the thermal effects. Although IRE generally considered as a non-thermal treatment, our result showed that the temperature in bovine liver tissue increased mainly in the range of 37–50 °C and can reach up to 72 °C in the high voltage (3000 V) parameter. Similar to the result from Agnass et al. [26], IRE can be considered as a mild-hyperthermic treatment, instead of non-thermal treatment.

Two-dimensional models were built instead of 3D models for the sake computational speed and based on the assumption that the electric and temperature distributions are symmetric. The models simulate two needle electrodes in the real IRE setup and still considered a good approximation for the planning of IRE treatments [16]. In addition, the 2D plane considered to represent the experimental setup consisted in a cross section perpendicular to the electrodes along the z-axis.

The models were validated based on experimental measurements of temperature. Validation was successfully achieved, where a great part of the experiments presented percentage errors lower than the assumed threshold of 10%. However, some measurements presented relatively high errors. A possible source of error might be related to the insertion of the temperature probes in a wrong place. The bovine liver tissue has the disadvantage of not being a transparent material. This lack of visual information makes it difficult to make sure that the temperature probes were positioned at the correct locations.

**Fig. 7:**
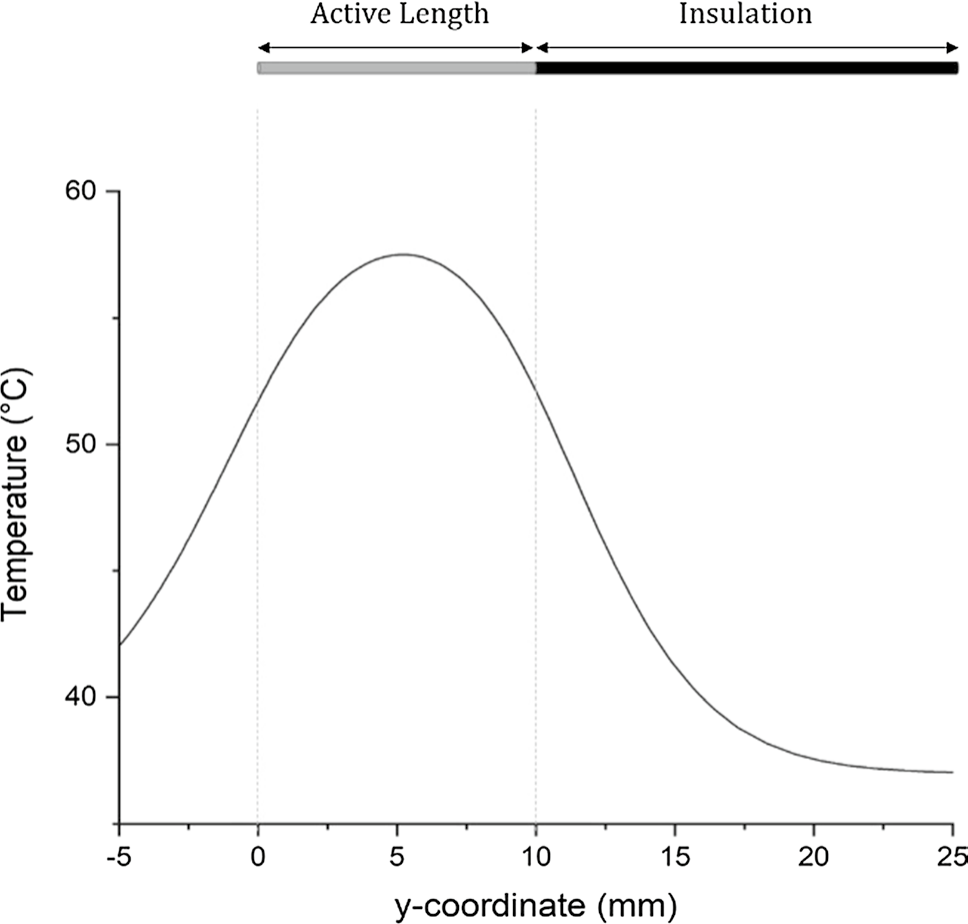
Temperature distribution along a vertical cut line at the center point between electrodes

Temperature measurement error due to a wrong probe placement can be illustrated using temperature distribution in Fig. 7. A *y*-coordinate of 0 mm represents the point of the vertical line that is aligned with the tip of the electrodes. *y*-coordinate values between 0 and 10 mm correspond to the portion of the vertical line that is aligned with the active part of the electrodes. As a result, the center measurement point is located at the y-coordinate of 5 mm. This figure shows that the temperature distribution along the vertical line is not constant. By having a small error in the probe location, the temperature measurement can have a different result. Therefore, it is suggested to have a visual feedback for the probe position, such as live image from ultrasound, to ensure that the probe is inserted at the right place.

### Conclusion and future research

The approach presented in this study enables the optimization of the IRE parameter using validated models. An optimized combination of IRE parameters for bovine liver was achieved by maximizing the electric field between the electrodes while limiting the maximum temperature in the tissue at 50 °C. As a result, an ablation area of 250.82 mm^2^ and a maximum temperature of 45.36 °C were the measured responses by inserting two needle electrodes with an active length of 10 mm, separated by 10 mm, and the delivery of 50 pulses with a width of 41.21 µs and amplitude of 3000 V. No thermal damage in bovine liver tissue is expected to occur after using this IRE configuration and, consequently, it could be applied in a clinical context.

Apart from five different parameters that were studied, there are other parameters that can also be investigated. One of them is the implementation of breaks between pulses. There is evidence that the rest periods between sequences of pulses can contribute to a decrease of the resultant temperature in the tissue [27, 28]. This can be an important parameter to be considered for optimization regarding the thermal effects of IRE. Another parameter is the waveform of the delivered pulses. It would be interesting to assess and compare the temperature outcomes from the application of exponential pulses and square pulses [29].

Using another algorithm, such as nondominated sorting genetic algorithms II (NSGA-II), to calculate the optimal IRE protocol would add valuable information to the optimization process [30]. Considering both signal and electrode parameter in the IRE treatment planning, results regarding the efficacy of the treatment could be improved and, therefore, it also is recommended to be an object for future research.
